# Retinal Nerve Fiber Layer Measures and Cognitive Function in the EPIC-Norfolk Cohort Study

**DOI:** 10.1167/iovs.16-19067

**Published:** 2016-04-19

**Authors:** Anthony P. Khawaja, Michelle P. Y. Chan, Jennifer L. Y. Yip, David C. Broadway, David F. Garway-Heath, Robert Luben, Shabina Hayat, Fiona E. Matthews, Carol Brayne, Kay-Tee Khaw, Paul J. Foster

**Affiliations:** 1 Department of Public Health and Primary Care, Institute of Public Health, University of Cambridge School of Clinical Medicine, Cambridge, United Kingdom; 2 NIHR Biomedical Research Centre, Moorfields Eye Hospital NHS Foundation Trust and UCL Institute of Ophthalmology, London, United Kingdom; 3 Division of Genetics and Epidemiology, UCL Institute of Ophthalmology, London, United Kingdom; 4 Department of Ophthalmology, Norfolk & Norwich University Hospital, Norwich, United Kingdom; 5 MRC Biostatistics Unit, Institute of Public Health, Cambridge, United Kingdom

**Keywords:** cognition, retina, epidemiology, biological markers, optic disk

## Abstract

**Purpose:**

We examined the relationship between retinal nerve fiber layer (RNFL) thickness and cognitive function in a population of older British adults.

**Methods:**

Participants of the European Prospective Investigation of Cancer (EPIC) Norfolk cohort study underwent ophthalmic and cognitive assessment. Measurements of RNFL thickness were made using the Heidelberg Retina Tomograph (HRT). Cognitive testing included a short form of the Mini-Mental State Examination (SF-MMSE), an animal naming task, a letter cancellation task, the Hopkins Verbal Learning Test (HVLT), the National Adult Reading Test (NART), and the Paired Associates Learning Test. Multivariable linear regression models were used to assess associations of RNFL thickness with cognitive test scores, adjusted for age, sex, education level, social class, visual acuity, axial length, and history of cataract surgery.

**Results:**

Data were available from 5563 participants with a mean age of 67 years. A thicker HRT-derived RNFL thickness was associated with better scores for the SF-MMSE (0.06; 95% confidence interval [CI], [0.02, 0.10], *P* = 0.005), HVLT (0.16, 95% CI [0.03, 0.29]; *P* = 0.014), and NART (−0.24, 95% CI [−0.46, −0.02], *P* = 0.035). The associations of RNFL thickness with SF-MMSE and HVLT remained significant following further adjustment for NART.

**Conclusions:**

We found a significant association between HRT-derived RNFL thickness and scores from cognitive tests assessing global function, recognition, learning, episodic memory, and premorbid intelligence. However, the associations were weak and not currently of predictive value. Further research is required to confirm and clarify the nature of these associations, and identify biological mechanisms.

Cognition refers to mental activity including perception, planned action, and thought.^1^ Dementia is a decline of cognitive function severe enough to interfere with social function, not associated with alteration of consciousness.^2^ Alzheimer's disease (AD) is the most common form of dementia.^2^ It has been estimated that there were 24.3 million people with AD worldwide in 2001, and that this will increase to 42.3 million in 2020, and 81.1 million by 2040.^3^ While there is no definitive therapy for AD, many experts believe that better characterization of potential precursor syndromes is important, and provides a target for possible preventative intervention in the future.^4,5^ Definitive diagnosis of AD is possible only at postmortem, the two main pathologic hallmarks being amyloid plaques and neurofibrillary tangles.^6^ Practically, a clinical diagnosis of AD usually is made, based on a careful history from the patient or caregiver, and neuropsychologic testing. Neuroimaging^7^ (including functional imaging^8^) and cerebrospinal fluid analysis^9^ may help identify or confirm cases of AD, and may form part of research diagnostic criteria.^10^ However, optimal methods and thresholds for these biomarkers currently are unknown^6^ and these tests are invasive or expensive, which limits their practical use on a large scale in the community. Identification of an affordable, noninvasive and acceptable biomarker for cognitive function may aid the diagnosis of AD or its precursor syndromes at a population level.

The optic nerve, unlike other cranial nerves, is surrounded by myelin produced by oligodendrocytes (rather than Schwann cells found in peripheral nerves), is encased by the meninges, and has the same embryonic origins as the central nervous system (CNS).^11^ Therefore, the optic nerve is considered part of the CNS, and as such, is the only directly visible part of it. Accurate measurements of the optic nerve head and surrounding retinal nerve fiber layer (RNFL) are now possible with modern ophthalmic imaging devices.^12^ It is an attractive hypothesis that these measurements might correlate with CNS structure or function, and that ocular imaging may provide an easy and noninvasive ancillary examination for the diagnosis of conditions, such as AD.^13,14^ Retinal nerve fiber layer thickness, as measured by the Glaucoma Diagnosis device with Variable Corneal Compensation (GDxVCC), was shown to be associated with cognitive function in healthy individuals from a genetically isolated population in the Netherlands.^15^ We have reported previously Heidelberg Retina Tomograph (HRT) measured RNFL thickness to be associated with educational attainment in the European Prospective Investigation of Cancer (EPIC)–Norfolk Eye Study,^16^ which might suggest a potential link with cognitive function.

The aim of this study was to examine the association of HRT derived RNFL measures with cognitive function in the EPIC-Norfolk cohort.

## Methods

EPIC-Norfolk is one of the UK arms of a pan-European prospective cohort study; detailed methods have been published elsewhere.^17^ In brief, 25,639 participants aged 40 to 79 years were recruited via general practices and examined between 1993 and 1997. Since virtually all residents in the United Kingdom are registered with a general practitioner through the National Health Service (NHS), general practice lists serve as population registers. Detailed ophthalmic examination and testing of cognitive function formed part of the third health examination of EPIC-Norfolk.^18,19^ In total, 8623 participants attended this examination between 2004 and 2011. EPIC-Norfolk was carried out following the principles of the Declaration of Helsinki and the Research Governance Framework for Health and Social Care. The study was approved by the Norfolk Local Research Ethics Committee (05/Q0101/191) and East Norfolk & Waveney NHS Research Governance Committee (2005EC07L). All participants gave written, informed consent.

### Ocular Measurements

All participants were examined in the research clinic and measurements were undertaken without pupil dilation by trained nursing staff following standard operating procedures, as detailed previously.^19^

Measurements of the RNFL were made using the HRT II (Heidelberg Retina Tomogram II; Heidelberg Engineering, Heidelberg, Germany). The HRT creates 3-dimensional images of the optic nerve head using scanning laser ophthalmoscopy and RNFL measurements are derived from the height of the retina at the disc margin.^20^ Measurements using the HRT II were taken after entering the participant's keratometry and refraction. If the HRT image quality was poor (topography standard deviation >40 μm) a repeat scan was undertaken. Contours around the disc margins were drawn manually and subsequently checked by an ophthalmologist (and redrawn if necessary). The HRT software subsequently was updated to Glaucoma Module Premium Edition (software version 3.1) and data exported following this. These derived data that are equivalent to HRT3-derived parameters. Only scans with a topography standard deviation ≤40 μm were included in analyses. We considered the “mean RNFL thickness” parameter which we have reported previously to be associated with educational attainment.^16^

Axial length was measured using a Zeiss IOLMaster Optical Biometer (Carl Zeiss Meditec Ltd, Welwyn Garden City, UK). Five measurements were taken per eye and a mean value calculated. Monocular visual acuity (VA) was measured using a logMAR chart (Precision Vision, LaSalle, IL, USA) on a light box under standard illumination. The test was performed with the aid of the participant's usual distance correction at 4 m (or 2 m, then 1 m if unable to read any letters). The test was terminated when the participant was able to read ≤3 letters on a line. The presenting VA of the better eye was considered in analyses.

### Cognitive Assessment

A battery of cognitive tests was done as part of the EPIC-Norfolk third health examination.^21^ The tests included a short-form of the Mini-Mental State Examination (SF-MMSE), an animal naming task, a letter cancellation task, the Hopkins Verbal Learning Test (HVLT), the National Adult Reading Test (NART), and the Cambridge Neuropsychological Test Automated Battery Paired Associates Learning Test (PAL).

The MMSE is a widely used and studied screening measure of cognitive impairment that contains 20 items with a potential total score of 30.^22^ The SF-MMSE was administered in EPIC-Norfolk, containing 11 items instead of 20, with a total possible score of 15.^23^ The rationale for this is that some of the items on the full MMSE may not be discriminating in a healthy population with a low prevalence of cognitive impairment.^24^ The full MMSE was designed for clinical samples, not for healthy population screening, and it is likely that some items will be answered correctly by most participants.^25^ This being the case, a shorter test without the easiest items would be quicker to administer, and potentially nearly as discriminating.^26^ The SF-MMSE has been shown to discriminate similarly to the full MMSE in another large population-based study.^23^ The SF-MMSE assesses memory recall, attention and calculation, object naming, registration, verbal registration, language, and visual-spatial/constructional performance.

The animal naming task involved recording the number of animals a participant can recall in 1 minute, which tests semantic verbal fluency,^27^ an important part of the assessment of patients with suspected dementia.^28,29^ The animal naming task has performed well at discriminating individuals with AD from those with normal cognitive function^30,31^ and predicted incident AD in an epidemiologic sample from Canada.^32^

The letter cancellation task^33^ involved a visual search of a grid of 780 random letters with the aim of crossing out as many target letters (the letters P and W, of which there were a possible 72) within 1 minute. The accuracy score (PW-accuracy) is the number of target letters correctly identified minus the number of target letters missed, and is a measure of attention and concentration.

The HVLT^34^ required participants to memorize a 12-item word list, composed of four words from 3 semantic categories (precious stones, human shelter, and animals with four legs). The words were displayed on a computer screen in very large font with an interval of 1 second between each word presentation. At the end of the presentation, the participant was asked to recall as many of the words as possible. The list was presented a further two times. The total score for the three HVLT trials was considered, with a maximum possible score of 36.

A shortened version of the NART^35^ required participants to correctly pronounce a set of 25 words of varying difficulty that do not follow the usual grapheme-phoneme and stress rules of pronunciation. These “irregular” words can be read correctly only if the participant recognizes them in their written form. The words were displayed in very large font on a computer screen, with the participant in control of the pace of moving to the next word. A score out of 50 was derived, as described previously.^35^ A lower NART score indicates a better cognitive performance.

The PAL assessed episodic memory and new learning, and has been shown to be very sensitive at detecting memory deficit in the very early stages of dementia.^36–38^ Boxes were displayed on a touch screen and opened in a random order to reveal a distinct pattern in one or more boxes. After all the boxes were opened, the pattern(s) were displayed in the middle of the screen one at a time and the participant required to touch the box which contained the pattern. There were 8 stages to the test and up to 10 repeat “reminder” presentations of the pattern locations. The total number of errors made, adjusted for the number of stages completed, was used in the present analysis, with a lower PAL score indicating a better cognitive performance.^39^

Social class and educational level were ascertained at the first health examination. Social class was recorded according to the Registrar-General's occupation-based classification system and was based on the participant's last occupation if they were retired. Educational level was recorded and classified into four groups according to the highest qualification achieved.

### Statistical Analysis

We considered the mean RNFL thickness of the right and left eyes of each participant if good quality data from both eyes were available. If only one eye had good quality data, that eye was considered. Data for PAL were log-transformed to derive a normal distribution. Mean and standard deviations for each of the cognitive test scores were calculated for the whole cohort and for quartiles of HRT mean RNFL thickness. A test of trend across the quartiles for each cognitive test was done. Potential confounders were decided a priori. Despite the large font on test displays, visual acuity may influence performance on cognitive tests as well as be related to RNFL thickness. Axial length is known to be associated with RNFL thickness^16,40^ and education level.^41^ A history of cataract surgery has been associated with RNFL measurements,^40^ and it might be that whether participants seek care for cataracts or not is related to cognitive function. Disc area has been shown to be associated with several HRT parameters, and variation in this parameter is thought to be largely physiologic rather than pathologic.^16^ To assess the effect of possible confounders on the association between RNFL measures and cognitive function, we used multivariable linear regression models with the cognitive score as the dependent variable and the RNFL measure together with covariables as the explanatory variables. We used three types of multivariable model: adjustment for disc area, adjustment for disc area and age, and adjustment for disc area, age, sex, educational level, social class, visual acuity of the better eye, axial length, and a history of cataract surgery (“maximally adjusted”). Regression coefficients were calculated per standard deviation change of HRT RNFL thickness. Stata version 13.1 (StataCorp LP, College Station, TX, USA) was used for all statistical analyses.

## Results

There were complete data for HRT RNFL thickness, cognitive tests and covariables from 5563 participants with a mean age of 67 years (range, 48–89); 56% were women. Compared to participants who attended the EPIC-Norfolk Eye Study but were excluded from analyses due to missing or poor quality data (*n* = 3060), included participants were significantly younger (*P* < 0.001) and had higher educational attainment (*P* < 0.001). Included and excluded participants did not differ significantly by sex (*P* = 0.06) or social class (*P* = 0.17).

Mean scores for the cognitive tests in the whole cohort, and also by HRT RNFL thickness quartiles are presented in Table 1. There were statistically significant trends for a better cognitive test score with thicker RNFL quartile for all cognitive tests.

**Table 1:**
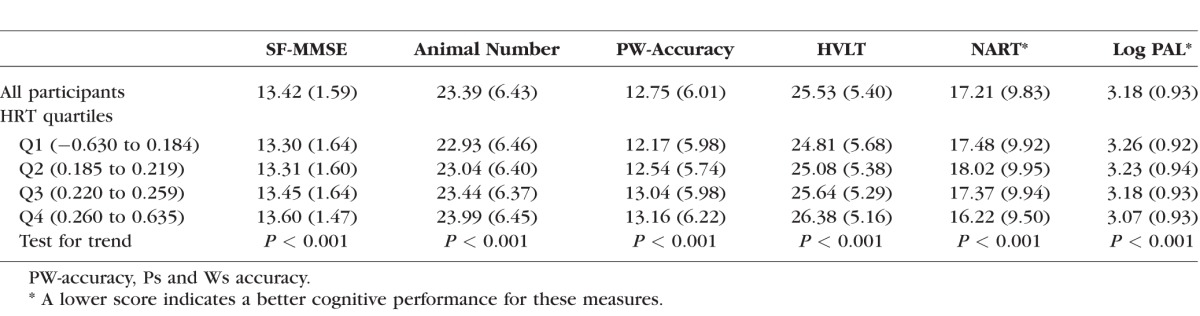
Mean (SD) Cognitive Scores for All Included Participants (*n* = 5563) and by Quartiles of HRT RNFL Thickness

Table 2 presents results from multivariable regression models with progressive adjustment for possible confounders. A thicker HRT RNFL measurement was associated with a better score for all cognitive tests in analyses adjusted for disc area only. Following adjustment for age and further adjustment for all covariables, significant associations remained between a thicker HRT RNFL measure and better cognitive scores for SF-MMSE, HVLT, and NART tests. We also examined the associations of HRT RNFL thickness with SF-MMSE and HVLT further adjusted for the NART score (which may represent early life cognitive function rather than later cognitive change); the associations remained statistically significant (SF-MMSE: 0.05, 95% confidence interval [CI; 0.01, 0.09], *P* = 0.015; HVLT: 0.14, 95% CI [0.01, 0.27], *P* = 0.036).

**Table 2:**
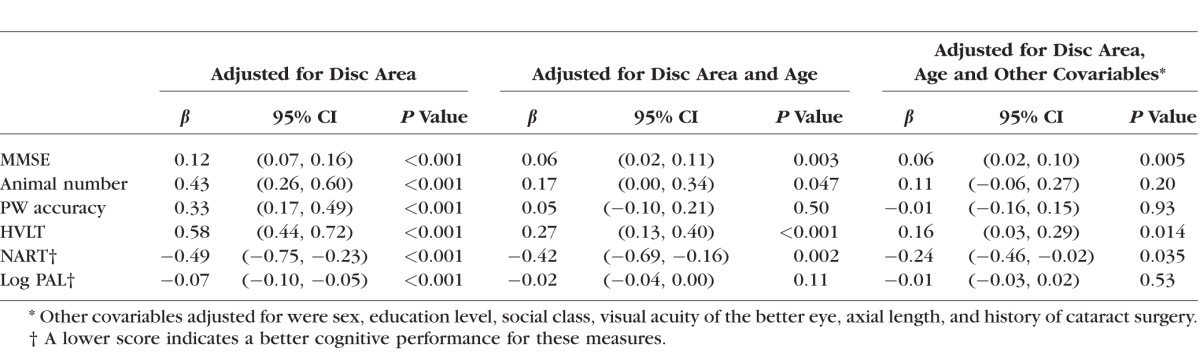
Regression Coefficients per SD Change in HRT RNFL Thickness, With Cognitive Scores as the Dependent Variables

The adjusted *R*^2^ for the maximally adjusted models were 0.092 for SF-MMSE, 0.214 for HVLT, and 0.149 for NART. The adjusted *R*^2^ for the models only adjusted for disc area were 0.006 for SF-MMSE, 0.011 for HVLT, and 0.002 for NART.

## Discussion

In this population-based sample, we found thinner HRT-derived mean RNFL thickness measures to be associated with poorer performance for three tests (SF-MMSE, HVLT, and NART) which assess several domains of cognitive function. The SF-MMSE is a global measure of cognitive function,^23^ while the HVLT assesses recognition, learning, and episodic memory.^34^ The NART is an estimate of premorbid intelligence.^35^ These associations remained following statistical adjustment for possible confounders, including age.

The poorer cognitive performance we observed in participants with thinner HRT RNFL measurements may have a few different explanations. The association may be due to a thinner RNFL measure reflecting poorer structural health of the CNS and a greater degree of neurodegeneration, which in turn might be related to a poorer cognitive function. If this is the case, further refining of which attributes of RNFL structure best relate to cognitive function would be of interest. As it stands, the association we observed was statistically significant but weak; HRT RNFL thickness alone explained no more than 1% of the variability of the cognitive scores and, therefore, would be of limited predictive utility for cognitive function. Our findings therefore, at present, do not support the concept that HRT RNFL measures may act as a biomarker for cognitive health.

Other possible explanations for our observed association between HRT measured RNFL thickness and cognitive function include residual confounding by either age or axial length. Cognitive function^42^ and RNFL thickness^16,40^ are known to decline with age. It is possible that linear adjustment for age did not completely remove the confounding effect of age for the association between HRT RNFL thickness and cognitive function. However, results were similar following further adjustment for the square of age (data not shown).

Longer axial length is associated with higher educational attainment^41^ and this in turn may be associated with better cognitive function). There is conflicting evidence regarding how RNFL thickness varies with axial length. We have previously reported thinner HRT-measured RNFL in longer eyes using data from the present cohort.^16^ Therefore, confounding by axial length is unlikely to explain an association between HRT RNFL thickness and cognitive function in our study population. Furthermore, additional adjustment for the square of axial length did not significantly alter our results (data not shown).

Five of the six cognitive tests examined in this study required some visual input for successful completion (all except the animal naming task). It is possible that thinner RNFL is reflecting poorer vision and, therefore, worse performance on cognitive tests requiring vision. However, the prevalence of central visual impairment measured using logMAR charts was very low in this cohort,^19^ and very large font size was used for the words presented in the tests we found significant associations with. Furthermore, we adjusted analyses for VA. Therefore, it seems unlikely that the association we observed between RNFL thickness and cognitive function was explained entirely by variation in visual function. It is unclear why we detected associations between RNFL thickness and only three of the six cognitive measures. It is possible that only cognitive decline in certain domains is biologically associated with changes in the RNFL. A more likely explanation is that our study was underpowered to consistently detect small associations for all the cognitive tests.

It should be noted that while the HRT derived parameter is termed “mean RNFL thickness,” it does not represent true RNFL thickness in the same way as optical coherence tomography (OCT). The parameter is derived from the height of the retina above a reference plane at the disc margin, and, therefore, can be considered a proxy measure rather than a true anatomic measurement of the RNFL. Therefore, other attributes of optic nerve head anatomy may be driving the relationship with cognitive function than RNFL thickness per se. For example, temporal tilt of the optic disc may result in higher measures of HRT-derived RNFL thickness, and optic disc tilt may be associated with cognitive function (though this is not known).

Our study population included participants with glaucoma and we purposely did not exclude these participants as they may contribute important signal for the relationship between optic nerve head measures and cognitive function. We hypothesize that a primary neurodegenerative process may contribute to cognitive decline and the etiology of primary open-angle glaucoma. We conducted further analyses to determine if the association between RNFL thickness and cognitive function remained following exclusion of participants with glaucoma. There were 171 participants with a history of either glaucoma medication use (*n* = 143) and/or glaucoma surgery (*n* = 41). Following exclusion of these participants, we repeated the analyses presented in Table 2 and yielded similar results (Supplementary Table). The cognitive scores were distributed normally in our population except for PAL and SF-MMSE. The distribution for PAL was skewed and, therefore, we analyzed log-transformed data as presented in this study. The distribution for SF-MMSE displayed a ceiling effect and was not amenable to transformation. As an additional analysis, we carried out a logistic regression analysis for SF-MMSE as a binary outcome variable (defined by the median value: participants scoring < 14 versus participants scoring ≥ 14); the maximally adjusted association remained highly significant (odds ratio per SD change in RNFL thickness, 1.10; 95% CI [1.04, 1.17]; *P* = 0.002).

There are two other published reports examining the relationship between RNFL measures and cognitive function in healthy populations.^15,43^ Our findings, in part, are in agreement with results from a similar study of 1485 individuals from the Erasmus Rucphen Family (ERF), which also reported thinner RNFL to be associated with poorer scores for several different cognitive tests.^15^ In agreement with our study, the effect estimates detected in the ERF study were small suggesting poor predictive value. In a small OCT study of 96 participants from the Lothian Birth Cohort 1936, thicker inferior RNFL was associated with better general processing speed (a principal component of several cognitive test scores) after adjusting for age and sex.^43^ Following further adjustment for IQ score at age 11, several more significant associations between OCT RNFL parameters and cognitive components were found, but in a direction consistent with better cognitive functioning in participants with thinner RNFL.^43^ To our knowledge, our study is the largest study to date examining the association between RNFL measures and cognitive function in healthy adults, and in contrast to the ERF study, our cohort sampled the general population rather than an in-bred population.

Further evidence for an association between RNFL measures and cognitive function comes from studies demonstrating thinner RNFL in patients with AD or mild cognitive impairment compared to controls, as measured by HRT^44^ or OCT.^45–48^ Furthermore, there also is histopathologic evidence of optic nerve and retinal changes in AD. Studies involving postmortem examination of eyes from patients with AD have found significant differences in comparison with eyes from age-matched controls.^49–51^ There also are several studies associating AD with glaucoma.^52–55^ A link between these two diseases would support the concept of an underlying generalized susceptibility to neurodegeneration causing the association between RNFL measures and cognition. The risk of incident dementia in 812 participants of the Three-City-Bordeaux-Alienor study was 4-fold higher in those diagnosed with glaucoma at baseline (odds ratio, 3.9; 95% CI, 1.5–10.4).^55^ Wostyn et al.^56^ have hypothesized that the association between AD and glaucoma is causal, mediated by a lower cerebrospinal fluid pressure.

Strengths of the present study include the large sample size and the population-based design. There are several limitations of our study. The number of participants included in the current study represent a relatively small subset of the original baseline cohort, largely because the cognitive and ophthalmic assessment occurred at the third health examination which began over 10 years after baseline examination; this may induce a survivor bias. Participants excluded from analyses were older and less educated than included participants; the effect of this would be to truncate distributions and reduce statistical power to detect associations unless the associations are in the opposite direction for those excluded, which is unlikely. Our data are cross-sectional, and, therefore, it is not possible to assess whether RNFL measures can predict future cognitive decline from this study. Arguably, prediction of future cognitive decline would be most of interest, and examining this may become a future study as the current cohort continues follow-up. Another limitation is that the HRT is becoming used less commonly in routine clinical assessment of ophthalmic patients with the increasing popularity of OCT.^57^

In summary, we found significant associations between HRT-derived RNFL thickness and several domains of cognitive function. While the strength of association limits any predictive value of HRT measures for cognitive function at present, these findings suggest further study of the relationship between retinal neuroanatomy and cognitive function is worthwhile.

## Supplementary Material

Supplement 1
