# Supplementary material for: Retinal Nerve Fiber Layer Measures and Cognitive Function in the EPIC-Norfolk Cohort Study
Source: Invest Ophthalmol Vis Sci. 2016 Apr 19;57(4):1921–6. doi: 10.1167/iovs.16-19067 (PMC4849871; doi:10.1167/iovs.16-19067)
Supplement: Supplement 1 [file i1552-5783-57-4-1921-s01.pdf]

Supplementary Table: Results following exclusion of participants with glaucoma. Regression coefficients are presented per standard deviation (SD) change in HRT RNFL thickness, with cognitive scores as the dependent variables.

	Adjusted for disc area			Adjusted for disc area & age			Adjusted for disc area, age & other covariables**		
	β	95% CI	P-value	β	95% CI	P-value	β	95% CI	P-value
**MMSE**	0.11	(0.06, 0.15)	**<0.001**	0.06	(0.02, 0.10)	**0.008**	0.05	(0.01, 0.09)	**0.013**
**Animal number**	0.43	(0.25, 0.60)	**<0.001**	0.18	(0.01, 0.35)	**0.038**	0.11	(-0.05, 0.28)	0.18
**PW accuracy**	0.32	(0.16, 0.49)	**<0.001**	0.06	(-0.10, 0.22)	0.47	0.00	(-0.16, 0.16)	0.99
**HVLT**	0.54	(0.40, 0.68)	**<0.001**	0.23	(0.10, 0.37)	**0.001**	0.13	(-0.00, 0.26)	0.056
**NART***	-0.51	(-0.78, -0.25)	**<0.001**	-0.44	(-0.71, -0.17)	**0.001**	-0.24	(-0.46, -0.02)	**0.036**
**Log PAL***	-0.07	(-0.10, -0.05)	**<0.001**	-0.02	(-0.05, 0.00)	0.08	-0.01	(-0.03, 0.01)	0.41

SF-MMSE – short-form Mini-Mental State Examination; PW-accuracy – Ps and Ws accuracy; HVLT – Hopkins Verbal Learning Test; NART - National Adult Reading Test; PAL - Paired Associates Learning Test.

* a lower score indicates a better cognitive performance for these measures.

** other covariables adjusted for were sex, education level, social class, visual acuity of the better eye, axial length and history of cataract surgery.
